# Prevalence of vancomycin-resistant *Enterococci* in India between 2000 and 2022: a systematic review and meta-analysis

**DOI:** 10.1186/s13756-023-01287-z

**Published:** 2023-08-21

**Authors:** Emily Smout, Navaneethan Palanisamy, Sabeel P Valappil

**Affiliations:** Chester Medical School, University of Chester, Bache Hall, Countess View, Chester, CH2 1BR UK

**Keywords:** Vancomycin, Vancomycin-resistant *Enterococci*, Antimicrobial resistance, Prevalence

## Abstract

**Background:**

Vancomycin-resistant *Enterococci* (VRE) infections are recurrently reported in different parts of India in the last two decades. However, an up-to-date, countrywide information concerning the prevalence and the rate of VRE in India is limited and hence this study aimed to estimate the pooled prevalence of VRE in India.

**Methods:**

A literature search was performed using various databases. The Preferred Reporting Items for Systematic Reviews and Meta-Analyses (PRISMA) guidelines were followed throughout. Cross-sectional studies reporting the prevalence of VRE in India from human samples whereby at least two *Enterococci* were isolated between 1 January 2000 and 31 December 2022 were sought for inclusion. Data were extracted and analysed using Microsoft Excel and Comprehensive Meta-analysis version 4, respectively.

**Results:**

Nineteen studies were included in the analyses. A collective total of 3683 *Enterococci* isolates were examined, of which 368 were VRE strains. The pooled prevalence of VRE in India was calculated at 12.4% (95% CI: 8.6–17.5; *Q* = 189.69; *I*^*2*^ = 90.51%; *p* = < 0.001). *E. faecalis* was the most frequently isolated species (1450 [39.37%]) followed by *E. faecium* (724 [19.66%]). Amongst the VRE strains, *E. faecium* was the most prevalent (214 [58.15%]) followed by *E. faecalis* (134 [36.41%]). An upsurge in the rate of VRE infections was observed in India over time: VRE prevalence was estimated at 4.8% between 2000 and 2010 and 14.1% between 2011 and 2020.

**Conclusion:**

This study presents the most up-to-date information on the rate of VRE infections in India. Though lower than the findings for some less developed countries, VRE prevalence in India is notable and on the rise.

**Supplementary Information:**

The online version contains supplementary material available at 10.1186/s13756-023-01287-z.

## Introduction

Antimicrobial resistance (AMR) has emerged as one of the most predominant threats to human health in the world today. AMR occurs when microorganisms evolve resistance mechanisms to protect them from the effects of antimicrobial drugs. Resultantly, AMR jeopardises the treatment of infections, leading to increased morbidity and mortality.

AMR is an ever-growing concern and specifically, vancomycin-resistant *Enterococci* (VRE) are of increasing importance, such that in 2017, vancomycin-resistant *Enterococcus faecium* has been listed by the World Health Organization (WHO) as a high-priority pathogen requiring research and development of new antibiotics (https://www.who.int/news/item/27-02-2017-who-publishes-list-of-bacteria-for-which-new-antibiotics-are-urgently-needed). It has been estimated that 1.27 million deaths were directly attributable to antibiotic-resistant bacteria worldwide in 2019, and a further 3.68 million deaths were indirectly associated with bacterial AMR [1]. Of these fatalities, *Enterococcus faecium* (*E. faecium*) and *Enterococcus faecalis* (*E. faecalis*) were each responsible for between 100,000 and 250,000 incidents whereby vancomycin-resistant strains were accountable for a considerable proportion of this burden [1], although other VREs were also noted.

*Enterococci* are Gram-positive, catalase-negative, non-spore-forming, facultative anaerobic bacteria comprising the commensal microflora of the human intestinal tract, and less frequently the vagina and mouth [2]. Despite their usual commensalism, *Enterococci* are opportunistic pathogens and have been widely recognised as some of the most frequently isolated pathogens causing serious nosocomial infections [3–5]. Namely, urinary tract infections (UTIs), bacteraemia, endocarditis, and surgical site infections [6] in which *E. faecium* and *E. faecalis* are the most commonly implicated, though the *Enterococcus* genus consists of more than 50 species [7]. In addition to their infection-causing ability, *Enterococci* are renowned for their antibiotic-resistant nature, showing both inherent and acquired resistance to a vast range of antimicrobials typically used for treating Gram-positive bacterial infections including vancomycin [8].

Vancomycin is a glycopeptide antibiotic that works by inhibiting bacterial cell wall synthesis [9]. It has been used to fight infections caused by Gram-positive pathogens since the 1950s and was considered a last line of defence against multi-drug-resistant (MDR) bacteria [9]. However, isolates of *Enterococcus gallinarum* and *Enterococcus casseliflavus* exhibit intrinsic, low-level resistance to vancomycin with minimum inhibitory concentrations of up to 32 µg/mL [10]. On the other hand, *E. faecium*, *E. faecalis*, and numerous other *Enterococcus* species display acquired resistance to vancomycin [11]. Vancomycin resistance is obtained by these *Enterococci* via mutations and/or the gain of exogenous genetic material which confers resistance [11]. Various genes such as *vanA*, *vanB*, *vanD*, *vanE*, *vanG*, and *vanL* have been proven to contribute towards vancomycin resistance in *Enterococci* [11]. Clinical isolates of VRE were first discovered in 1986 in France by Leclercq et al. [12], and then in the UK in 1987 by Uttley et al. [13]; VRE have since spread globally and owing to their capability of AMR towards many clinically relevant antibiotics, they are acknowledged as a universal public health concern [14].

India has one of the highest burdens of infectious disease on the planet [15]. It is also ranked among the countries with the greatest AMR burden in both humans and animals [16]. The earliest recording of VRE in India was by Mathur et al. in 1999 [17]. Although there exist numerous studies reporting the prevalence of VRE in local areas of India, at the time this research was conducted, there had not yet been a comprehensive analysis investigating the national pooled prevalence of VRE. Therefore, this study aimed to evaluate and summarise the prevalence of VRE in India through a systematic review and meta-analysis using cross-sectional studies carried out in different parts of the country.

## Methods

### Literature search strategy

A comprehensive literature search using electronic databases was conducted to identify all the relevant published articles from 1 January 2000 to 31 December 2022. The databases explored were PubMed, Scopus, and Google Scholar. In the case of Google Scholar, it was used as a secondary supplementary search whereby only the first 100 articles were sought. Further, the reference lists of the included papers were scrutinised for any appropriate additional material.

Key terms used in the search included “prevalence”, “rate”, “frequency”, “epidemiology”, “epidemiological”, “cross-sectional”, “*Enterococci*”, “*Enterococcus*”, “*E. faecalis*”, “*E. faecium*”, “vancomycin resistance”, “vancomycin resistant”, “VRE”, “antimicrobial resistance”, “antimicrobial resistant”, “antibiotic resistance”, “antibiotic resistant”, “drug resistance”, “drug resistant”, and “India” in combination with suitable Boolean operators. Truncation was used wherever possible. See **Supplementary Table 1** for exact search inputs.

This search strategy was followed by all the authors independently to retrieve the appropriate articles. Sabeel P Valappil additionally acted as the referee to resolve any disagreements. All of the studies obtained were exported to the referencing software EndNote version X9. Referenced papers were screened for duplicates initially by EndNote and then manually; all duplicate articles were removed.

The Preferred Reporting Items for Systematic Reviews and Meta-Analyses (PRISMA) guidelines [18] were followed throughout.

### Eligibility criteria

Every article was considered suitable for inclusion if:

- Published in English.
- Published between (and including) 1 January 2000 and 31 December 2022.
- Conducted in India in any setting.
- Cross-sectional by design.
- The study subjects were human.
- No fewer than two *Enterococci* were isolated from samples.
- Prevalence of VRE was reported or the total number of VRE in addition to the total number of *Enterococci* isolates.

Whereas articles were excluded from this investigation if:

- No English translation had been published.
- Conducted outside of India.
- Studies were not cross-sectional.
- Non-human subjects were included in the study population i.e., animals, inanimate objects, the environment, water sources, etc.
- Studies had less than two *Enterococci* isolates.
- Antimicrobial susceptibility was reported in other organisms but not *Enterococci*.
- Antimicrobial susceptibility was tested for except vancomycin.
- No clear methods for detecting antimicrobial susceptibility were defined.
- Full texts were not accessible.
- Studies provided insufficient information.

### Literature screening

All of the studies yielded from the extensive literature search were independently evaluated against the eligibility criteria; titles and abstracts were screened first followed by the main text. Every paper which fulfilled the inclusion criteria were shortlisted for quality assessment.

### Quality assessment

The quality of all remaining studies was assessed using the Joanna Briggs Institute (JBI) critical appraisal checklist for studies reporting prevalence data [19] which contains nine questions. Articles that scored five or lower out of the nine criteria sections were deemed inadequate and not included in the meta-analysis. The nine questions are,

- Was the sample frame appropriate to address the target population?
- Were study participants sampled appropriately?
- Was the sample size adequate?
- Were the study subjects and the setting described in detail?
- Was the data analysis conducted with sufficient coverage of the identified sample?
- Were valid methods used for the identification of the condition?
- Was the condition measured in a standard, reliable way for all participants?
- Was there an appropriate statistical analysis?
- Was the response rate adequate, and if not, was the low response rate managed appropriately?

### Data extraction

Key information including authors’ names, year of publication, study period, study location (according to the six Zonal Councils of India), study population, specimen type(s), antimicrobial susceptibility testing (AST) method(s), the different *Enterococci* species isolated, the total number of *Enterococci* isolates (plus, the total number of individual *Enterococci* species if disclosed), and the total number of VRE isolates with VRE prevalence shown as a percentage (plus, the total number of individual VRE species if disclosed) was extracted from the eligible articles and recorded in Microsoft Excel.

If studies did not report the prevalence of VRE, it was obtained via manual calculation by dividing the number of VRE isolates by the number of *Enterococci* isolates and multiplying the answer by 100. Conversely, if studies did not report the total number of VRE isolates, it was procured by dividing the percentage VRE prevalence by 100 and multiplying the answer by the total number of *Enterococci* isolates.

### Statistical analysis

Computations were performed using Comprehensive Meta-Analysis version 4; where studies reported the total number of VRE isolates as zero, a continuity correction (adding 0.5) was applied. The random-effects model by DerSimonian and Laird was used to determine the pooled prevalence of VRE.

Heterogeneity was assessed using Cochran’s *Q* and the *I*^*2*^ tests. Sensitivity analysis using a one-study-removed technique was carried out to investigate the potential source of heterogeneity and evaluate the reliability of the pooled prevalence. Where the necessary data was available, subgroup analyses for VRE prevalence by study location (based on the six Zonal Councils of India: North, North-east, East, South, West, and Central), study period (studies conducted between 2000 and 2010, and between 2011 and 2020), AST method(s) (each respective AST used singly alongside a category for the use of multiple techniques), and specimen type(s) (all specimen types used individually with a category for the use of multiple different specimen types) were also done to address the presence of heterogeneity.

Funnel plots were drawn to qualitatively examine publication bias further to which Duval and Tweedie’s trim-and-fill method was applied to insert “missing” studies and estimate adjusted effect sizes in the instance of funnel plot asymmetry. Publication bias was quantitatively evaluated by Begg and Mazumdar’s rank correlation and Egger’s regression test whereby a *p*-value of < 0.05 for either calculation was considered statistically significant.

## Results

### Study selection

**Fig. 1:**
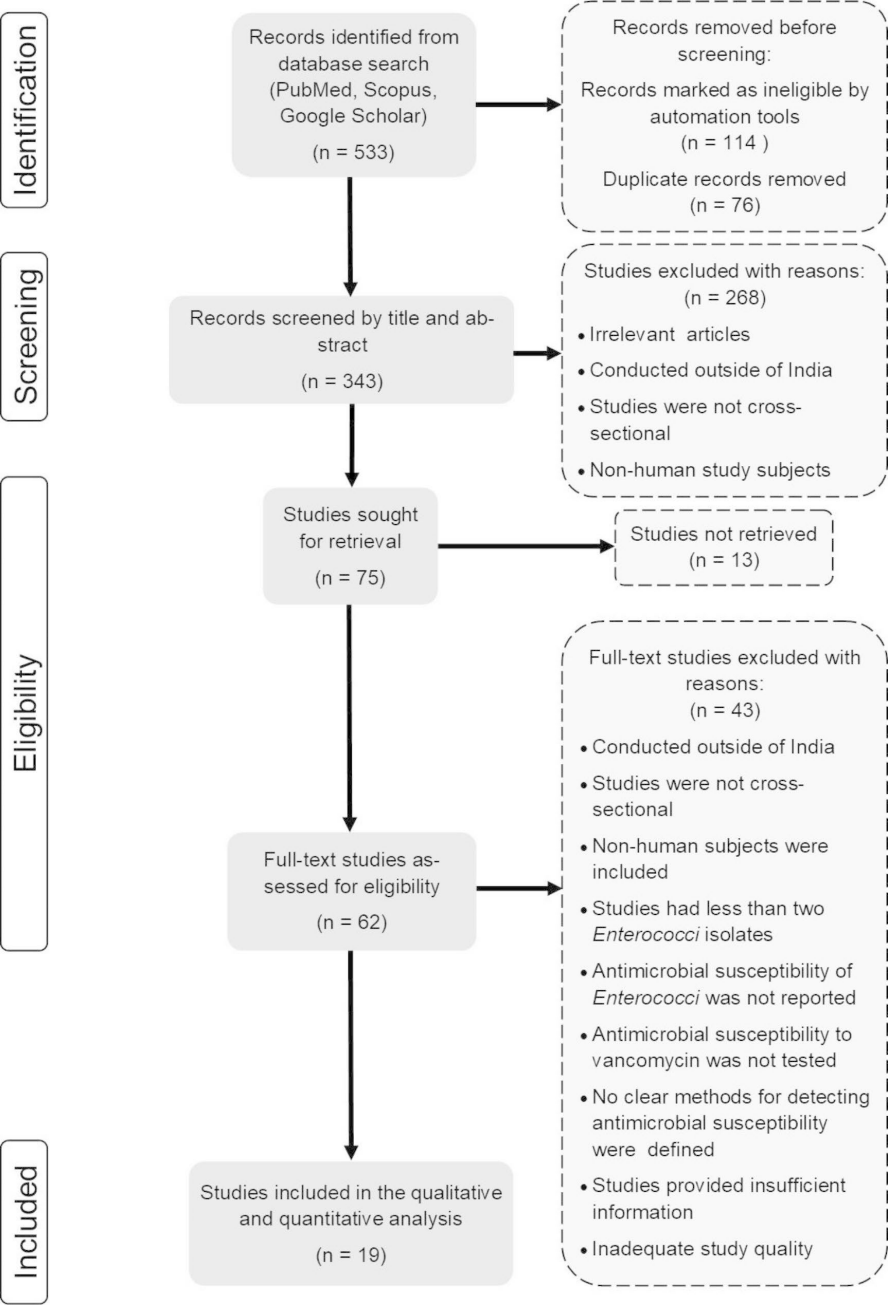
PRISMA flow chart of the literature search and study selection process

A PRISMA flow diagram delineating the results of the literature search and systematic study selection process is shown in Fig. 1. The database search yielded a total of 533 records: of which 62 full-text articles were reviewed for eligibility against the predetermined inclusion and exclusion criteria, followed by the quality assessment. Forty-three studies were justifiably eliminated, and the remaining 19 studies were incorporated into the meta-analysis. No suitable additional papers were acquired by searching reference lists. The quality assessment of the included studies is reported in **Supplementary Table 2**.

### Characteristics of included studies

Each of the studies included in the present research was cross-sectional by design. Of the 19 suitable articles, 18 were conducted in clinical settings. As per the Zonal Councils of India, seven studies were reported from Northern India, followed by three each from Western and Central India, two from both Southern and North-eastern India, and a single study was from Eastern India; one paper did not disclose a study location. All of the articles were published between 2004 and 2022. According to their respective study period: the earliest study began in October 2000 whereas the latest study finished in December 2020; four of the included papers did not disclose their period of study.

A collective total of 3683 *Enterococci* were isolated from various samples and examined for vancomycin resistance by several standard AST methods. Fourteen studies reported the number of *Enterococci* isolates to species level, and 16 studies reported the VRE to species level. It was found that 1450 (39.37%) *Enterococci* isolates belonged to *E. faecalis*, 724 (19.66%) belonged to *E. faecium*, and 95 (2.58%) belonged to other species (see **Supplementary Table 3**); 1414 (38.39%) isolates were not classified according to species. The prevalence of VRE ranged from 0.00 to 37.14%. Of the 368 VRE isolates, 214 (58.15%) were *E. faecium*, 134 (36.41%) were *E. faecalis*, 10 (2.72%) were other species, whilst the remaining 10 (2.72%) were only identified to genus level. Table 1 summarises the characteristics of the included studies.

**Table 1:** Characteristics of all studies included in the present analysis

Authors	Publication year	Study period	Study location by Zonal Council	Study population	Specimen type(s)	AST method(s)	Total *Enterococci* isolates	Total *E. faecalis* isolates	Total *E. faecium* isolates	Total VRE isolates (prevalence %)	*E. faecalis* VRE	*E. faecium* VRE
Bhargava et al. [56]	2022	November 2018 to December 2019	Central India	Patients > 5 years presenting with UTI	Urine	Kirby-Bauer disc diffusion	18	-	-	5 (27.78%)	-	-
Bhatt et al. [57]	2015	August 2011 to February 2014	-	In-patients and out-patients at a tertiary care hospital	Bile fluid, blood, CSF, pus, semen, tracheal aspirate, urine	Kirby-Bauer disc diffusion	200	150	50	28 (14.00%)	6	22
Das et al. [58]	2022	January 2016 to December 2018	Northern India	Patients symptomatic of UTI	Urine	VITEK-2, Kirby-Bauer disc diffusion	118	94	20	20 (16.95%)	14	6
Das et al. [59]	2021	January 2019 to December 2020	Eastern India	Patients ≥ 18 years developing UTI symptoms	Urine	VITEK-2	457	381	76	41 (8.97%)	18	23
Deshpande et al. [60]	2013	-	Western India	-	-	Broth microdilution	291	204	87	57 (19.59%)	38	19
Gangurde et al. [61]	2014	August 2012 to July 2013	Western India	-	-	Vancomycin agar screen test	180	108	58	15 (8.33%)	5	8
Goel et al. [62]	2016	April 2013 to May 2014	Northern India	Out-patients and in-patients < 48 h after hospital admission suspected of UTI	Urine	Kirby-Bauer disc diffusion, Vancomycin agar screen test, MIC by agar dilution	115	61	42	13 (11.30%)	6	5
Hazarika et al. [24]	2021	-	North-eastern India	Healthy individuals from Adi tribes	Stool	Kirby-Bauer disc diffusion	6	-	-	2 (33.33%)	-	-
Jain et al. [63]	2022	January 2014 to June 2015	Northern India	Pregnant women who underwent Caesarean delivery and developed surgical site infection within 30 days	Infected surgical site swab	Kirby-Bauer disc diffusion	9	-	-	3 (33.33%)	-	-
Kapoor et al. [64]	2005	April to October 2001	Northern India	Paediatric in-patients with bacteraemia	Blood	Vancomycin agar screen test, MIC by agar dilution	50	10	33	0 (0.00%)	0	0
Meena et al. [65]	2017	November 2015 to April 2016	Northern India	In-patients and out-patients symptomatic of UTI	Urine	Kirby-Bauer disc diffusion	70	9	61	26 (37.14%)	1	25
Phukan et al. [66]	2016	-	North-eastern India	In-patients and out-patients of a tertiary care hospital	Blood, pus, sputum, throat swabs, urine	Kirby-Bauer disc diffusion	67	54	13	16 (23.88%)	9	7
Praharaj et al. [67]	2013	November 2008 to October 2009	Southern India	In-patients and out-patients	CSF, peritoneal fluid, pleural fluid, pus, synovial fluid, tissue biopsies, urine, wound swabs	Kirby-Bauer disc diffusion, Vancomycin agar screen test, MIC by agar dilution	367	-	-	32 (8.72%)	29	0
Purohit et al. [68]	2017	October 2013 to April 2015	Northern India	Hospitalised patients	Blood, pus, urine	MIC by agar dilution, E test	250	82	162	57 (22.80%)	2	55
Sami et al. [69]	2020	March 2018 to February 2019	Central India	In-patients and out-patients of a tertiary care hospital	Pus, urine	Kirby-Bauer disc diffusion, VITEK-2	1014	-	-	31 (3.06%)	1	30
Shinde et al. [70]	2012	-	Western India	In-patients and out-patients of a tertiary care hospital	Blood, body fluids, pus, urine	Kirby-Bauer disc diffusion	54	47	5	0 (0.00%)	0	0
Sreeja et al. [71]	2012	January to December 2008	Southern India	-	Blood, body fluids, pus, tissue, urine	Kirby-Bauer disc diffusion, E test	128	97	31	0 (0.00%)	0	0
Taneja et al. [72]	2004	October 2000 to April 2001	Northern India	Patients suspected of UTI	Urine	Vancomycin agar screen test	144	80	17	8 (5.56%)	1	5
Yadav & Agarwal [73]	2022	June 2019 to May 2020	Central India	Patients requiring bacterial culture	Blood, body fluids, pus, urine	Kirby-Bauer disc diffusion	145	73	69	14 (9.66%)	4	9

*Note*: ‘-’ means data was not disclosed. AST = antimicrobial susceptibility testing, VRE = vancomycin-resistant *Enterococci*, UTI = urinary tract infection, MIC = minimum inhibitory concentration, CSF = cerebrospinal fluid

### Pooled prevalence of VRE

**Fig. 2:**
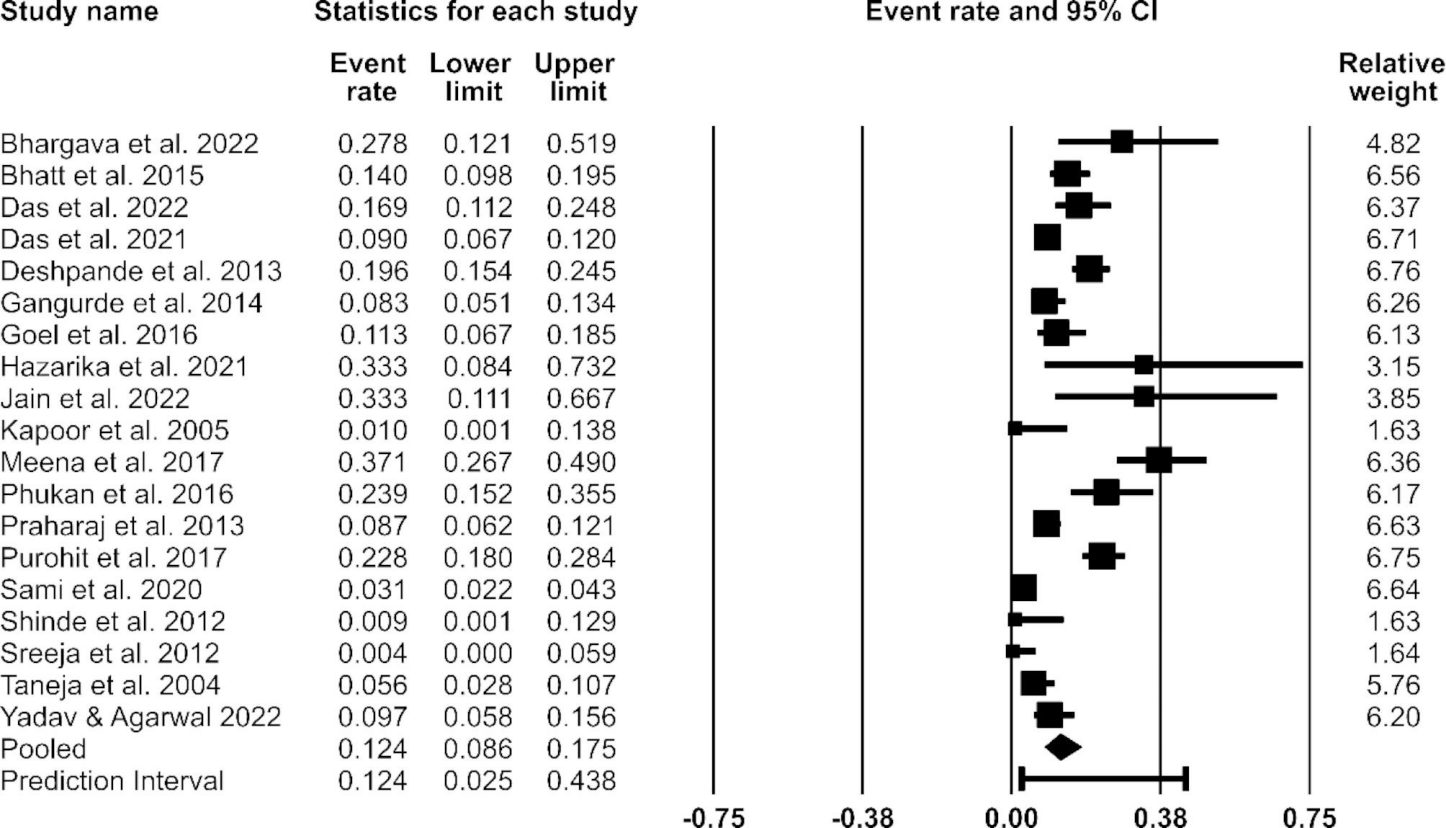
Forest plot showing the estimated pooled prevalence of vancomycin-resistant *Enterococci* (VRE) in India between 2000 and 2022. *Note*: The random-effects model was used. CI = Confidence interval

Based on the 19 selected studies, the pooled prevalence of VRE in India was estimated at 12.4% (95% confidence interval [CI]: 8.6–17.5) (Fig. 2). Significant heterogeneity (*Q* = 189.69; *I*^*2*^ = 90.51%; *p* = < 0.001) was observed.

### Sensitivity analysis

No single study significantly influenced the pooled VRE prevalence or the heterogeneity of the meta-analysis. The pooled prevalence of VRE in the sensitivity analysis (Table 2) ranged from 11.4 to 13.9% which lies within the 95% CI bounds of the overall pooled estimate. The degree of heterogeneity (*I*^*2*^) was between 84.49 and 91.03%.

**Table 2:** Sensitivity analysis of the included studies by stepwise study omission

Study omitted	Pooled prevalence	CI lower limit	CI upper limit	*Q*-value	*I*^*2*^ (%)	*p*-value
Bhargava et al. [56]	0.118	0.081	0.169	186.395	90.880	0.000
Bhatt et al. [57]	0.122	0.082	0.177	189.479	91.028	0.000
Das et al. [58]	0.121	0.082	0.174	187.939	90.955	0.000
Das et al. [59]	0.126	0.085	0.182	182.486	90.684	0.000
Deshpande et al. [60]	0.119	0.080	0.173	176.623	90.375	0.000
Gangurde et al. [61]	0.127	0.086	0.182	186.196	90.870	0.000
Goel et al. [62]	0.124	0.085	0.178	189.403	91.024	0.000
Hazarika et al. [24]	0.119	0.082	0.170	187.723	90.944	0.000
Jain et al. [63]	0.118	0.082	0.169	186.733	90.896	0.000
Kapoor et al. [64]	0.129	0.089	0.181	186.048	90.863	0.000
Meena et al. [65]	0.114	0.080	0.161	156.879	89.164	0.000
Phukan et al. [66]	0.118	0.081	0.169	182.637	90.692	0.000
Praharaj et al. [67]	0.126	0.085	0.182	183.352	90.728	0.000
Purohit et al. [68]	0.118	0.080	0.169	165.588	89.734	0.000
Sami et al. [69]	0.139	0.103	0.185	109.569	84.485	0.000
Shinde et al. [70]	0.129	0.090	0.181	185.838	90.852	0.000
Sreeja et al. [71]	0.130	0.091	0.183	183.066	90.714	0.000
Taneja et al. [72]	0.130	0.089	0.184	183.019	90.711	0.000
Yadav & Agarwal [73]	0.125	0.085	0.180	188.245	90.969	0.000

*Note*: The random-effects model was used. CI = Confidence interval

### Subgroup analysis

Subgroup analysis by Zonal Council regions of India revealed the highest combined prevalence of VRE was in North-eastern India (24.7%), followed by Northern India (16.3%), Western India (10.1%), Central India (9.2%), Eastern India (9.0%), and the lowest pooled prevalence of VRE was found in Southern India (2.6%). All analyses by location showed heterogeneity except for in North-eastern India (*I²* = 0.00%) but this category only comprised two studies thus making this finding inconclusive.

Analysis based on the study period showed a substantial increase in VRE prevalence in India over time: studies carried out between 2000 and 2010 had a pooled prevalence of 4.8% whereas studies conducted between 2011 and 2020 had a pooled prevalence of 14.1%. Heterogeneity was present in both subgroup analyses by study period; studies conducted between 2000 and 2010 showed less heterogeneity (*I²* = 64.0%) than studies done between 2011 and 2020 (*I²* = 93.0%) but the *I²* values for both subgroups were still high.

In the instance of AST method(s), pooled VRE prevalence was highest in studies that used only Kirby-Bauer disc diffusion (20.3%) while studies that used only the vancomycin agar screen test showed the lowest pooled prevalence (7.2%). High heterogeneity was observed in all AST method groups (wherever it was possible to assess) apart from the vancomycin agar screen test which had an *I²* value of 0.00% however this category consisted of just two studies and so this finding is indefinite.

Grouping by specimen types also revealed marked heterogeneity (*I²* = 89.71–93.82%). See Table 3 for further details.

**Table 3:** Subgroup analysis of the included studies

Subgroups	Number of studies	Total *Enterococci* isolates	Total VRE isolates	Pooled prevalence	CI lower limit	CI upper limit	*I²* (%)	*p*-value
*Zone* ^a^								
Northern India	7	756	127	0.163	0.096	0.264	85.425	0.000
North-eastern India	2	73	18	0.247	0.162	0.359	0.000	0.609
Eastern India	1	457	41	0.090	0.067	0.120	-	-
Southern India	2	495	32	0.026	0.001	0.363	80.067	0.025
Western India	3	525	72	0.101	0.038	0.241	86.533	0.001
Central India	3	1177	50	0.092	0.027	0.265	92.983	0.000
*Study period* ^b^								
2000–2010	4	689	40	0.048	0.021	0.108	64.047	0.039
2011–2020	11	2576	253	0.141	0.088	0.219	93.013	0.000
*AST method*								
Kirby-Bauer disc diffusion	8	569	94	0.203	0.123	0.315	79.360	0.000
VITEK-2	1	457	41	0.090	0.067	0.120	-	-
Broth microdilution	1	291	57	0.196	0.154	0.245	-	-
Vancomycin agar screen test	2	180	15	0.072	0.048	0.106	0.000	0.336
Multiple AST methods	7	2042	153	0.077	0.035	0.160	94.261	0.000
*Specimen type* ^c^								
Urine	6	922	113	0.151	0.082	0.259	89.708	0.000
Stool	1	6	2	0.333	0.084	0.732	-	-
Blood	1	50	0	0.010	0.001	0.138	-	-
Surgical site swab	1	9	3	0.333	0.111	0.667	-	-
Multiple specimens	8	2225	178	0.088	0.045	0.166	93.822	0.000

‘-’ means data was not available, CI = Confidence interval, AST = antimicrobial susceptibility testing, VRE = vancomycin-resistant *Enterococci*

*Note*: The random-effects model was used

^a^One paper did not report its study location

^b^Four studies did not address their respective study periods

^c^Two studies did not name the specimen type(s) used

### Publication bias

The drawn funnel plot (**Supplementary Fig. 1**) was asymmetric which indicated the presence of publication bias. When the Trim-and-Fill method was subsequently applied, one “missing” study was imputed to the right side of the mean effect (**Supplementary Fig. 2**). The corresponding adjusted estimate of VRE prevalence was 13.0% (95% CI: 9.05–18.37); close to the original pooled estimate of VRE in India.

The Egger’s regression test (intercept = -0.71; 95% CI: -3.99–2.58; *p* = 0.33) and Begg and Mazumdar’s rank correlation (*p* = 0.34) did not suggest significant publication bias.

## Discussion

In this report, we studied the prevalence of VRE in India through a systematic review and meta-analysis using cross-sectional studies carried out in different parts of the country between 2000 and 2022. We found that *E. faecalis* was the most commonly isolated *Enterococcus* species, followed by *E. faecium*; this finding corresponds with the results of a worldwide surveillance report on Gram-positive pathogens [20] and supports the idea that *E. faecalis* is the leading cause of Enterococcal infections (≤ 90%) [21]. However, this notion has long since been established and as demonstrated by Horner et al. [22] the proportion of infections caused by *E. faecium* has grown significantly in recent years to the extent that, in some cases, the number of *E. faecium* infections has exceeded the number *E. faecalis* infections. This suggests that *E. faecium* could become the dominant species causing Enterococcal infections in the future. As such temporal changes in the prevalence of *Enterococcus* species isolated from clinical specimens should be closely monitored.

Among the observed VRE isolates, our study found a higher frequency of *E. faecium* than *E. faecalis*. This outcome reinforces the understanding that *E. faecium* has a higher potential in the acquisition of resistance genes compared to *E. faecalis* [23] hence it is vancomycin-resistant *E. faecium* rather than *E. faecalis* listed as a priority 2 pathogen by the WHO in 2017.

The overall prevalence of VRE in India was estimated at 12.4% in the present study, based on the statistical analysis of 3683 *Enterococci* isolates accumulated from all six Zonal Council regions. Further, the time-related analysis showed an upsurge in the prevalence of VRE in the last decade: the estimated rate of VRE infections in India increased almost three-fold from 4.8% between 2000 and 2010, up to 14.1% between 2011 and 2020, also one study confirmed the presence of VRE in healthy individuals [24]. In comparison to similar investigations, our prevalence finding was considerably lower than the VRE frequency reported for Nigeria (26.5%) [25] and slightly less than the pooled prevalence of VRE determined for Ethiopia (14.8%) [26]. On the other hand, our pooled prevalence is higher than that of papers estimating VRE prevalence in the UK (9.8%) [27], Iran (9.4%) [28], and most notably across the continent of Asia (8.1%) [29] – in which the pooled prevalence of VRE in India and Pakistan (combined) was found to be 7.7%. While performing the subgroup analysis focusing on the study period, we considered the actual study period rather than the publication year of the article to obtain pooled prevalence. Shrestha et al. [29] too conducted subgroup analysis but used the publication years to group individual studies rather than their respective study periods. This is evident in the fact that Al-Talib et al. [30] carried out their work between September 2001 and June 2004 yet were categorised under the study period 2010 to 2020.

Various factors might explain the prevalence of VRE in India. One example is the lack of stringent adherence by healthcare personnel to infection control measures like hand hygiene. Findings of a multicentric observational study in 92 healthcare facilities across India showed that hand hygiene complete adherence rates were ≤ 38.3% whereby following all steps of handwash or hand-rub as suggested by the WHO was considered complete adherence [31]. Also, a survey study of hand hygiene practices among Indian medical undergraduates revealed that only 27.6% of students had good knowledge of hand hygiene practices [32]. Though it has been newly hypothesised that the prolonged use of hand hygiene products may cause AMR in healthcare settings [33], proper hand hygiene is regarded as one of the most important infection control measures [34, 35]. Hand hygiene is specifically referenced as a principal practice for the prevention and control of the dissemination of antibiotic resistance with proven effectiveness in the case of VRE transmission [36]. Therefore, by teaching and strictly enforcing correct hand hygiene measures in healthcare settings across India, VRE infections – as well as other healthcare-associated AMR infections – can be significantly reduced.

Another reason may be the excessive and injudicious use of antibiotics across India. As of 2010, India was the largest consumer of antibiotics for human health worldwide [37]. Further, a 2017 study by McGettigan et al. found that the overall sales of antibiotics in India were increasing and, in particular, the sales of Watch group antibiotics – such as vancomycin – were growing rapidly [38]. Later, Koya et al. calculated that India’s usage of broad-spectrum antibiotics classified under the Watch group list accounted for 54.9% of the total defined daily doses consumed [39]. Antibiotics are easily obtainable over the counter in most parts of the country [40]. This, coupled with a severe shortage of healthcare workers as well as unequal access to healthcare facilities, especially in more rural areas [41], has led to self-prescribing of antibiotics being a regular occurrence among many Indian citizens [42]. Additionally, inappropriate prescribing of antibiotics by some doctors has been noted [40]. For instance, Bose et al. [43] determined that in two tertiary centres in East India, 42.5% of vancomycin usage among hospitalised paediatric patients was improper. However, this study excluded all patients taking oral vancomycin thus making the calculation of unsuitable vancomycin usage a likely misestimation; though it is uncertain whether the result is an under- or overestimation. Despite this, India does not currently have a national-level antimicrobial stewardship plan [44]. Antimicrobial stewardship programs have demonstrated the ability to lower both antibiotic consumption and resistance; consequently, this has become a key strategy used to tackle AMR elsewhere [45]. By implementing a national antimicrobial stewardship program in India, the misuse and overuse of antibiotics such as vancomycin can be impeded, likely leading to a reduction in the frequency of VRE and AMR in general.

Lastly, it is feasible that the use of antimicrobials as growth promoters in animal husbandry has contributed to the prevalence of VRE in India. In 2010, India was the fourth largest consumer of antimicrobials for animal use worldwide [37]. Subsequent Bayesian projections predicted that India would contribute the largest relative increase in antimicrobial consumption for use within the livestock sector globally between 2010 and 2030 [46]. Excessive administration of antibiotics in food-producing animals can result in an accumulation of antibiotic residues in animal-derived products [47]. Such remnants could then be transmitted to people via the food chain and facilitate the transfer of antibiotic-resistance genes, thus enhancing AMR amongst humans. For example, avoparcin – an analogue of vancomycin – was widely used as an antibiotic growth promoter in many countries from the late 1970s [48] but was banned in all member countries of the European Union in 1997 as a result of studies reporting evidence of a causal link between the use of avoparcin and the occurrence of VRE in several food animals [49]. Following the discontinued use of avoparcin as a growth promoter, multiple European countries noted a significant decrease in the prevalence of VRE in livestock [50, 51] and also in healthy human individuals [51]. This proves that unreasonable use of antibiotics correlates directly with higher levels of antibiotic resistance within populations. In India, the presence of VRE in various animals intended for human consumption has been detected [52, 53]. Further, Preethi et al. [54] found a higher prevalence of VRE in conventionally raised Indian poultry chickens compared to organic chickens which suggests antimicrobial growth promoters might be a contributory factor. Nevertheless, there are, at present, no regulations in India regarding the use of antibiotics in food animals for domestic consumption; any existing guidelines apply only to certain types of seafood, and any food animals intended for exportation [55]. For that reason, it can be strongly hypothesised that by limiting the use of antimicrobials in animal husbandry, the frequency of VRE and other AMR organisms amongst food animals can be curtailed, and hence transmission of AMR to people via the food chain can be reduced too.

In the present study, an extensive literature search was done with explicit, pre-established inclusion and exclusion criteria. The statistical analyses performed were consistent with the standard statistical tests of any meta-analysis. Further, both heterogeneity and publication bias were explored. The included articles collectively examined a range of different specimens, and standard AST methods were used. Additionally, there was at least one eligible paper utilised from each of the six Zonal Council regions of India. Nevertheless, some constraints were evident in our investigation, particularly concerning the breadth and depth of data. First, pooled estimates of VRE prevalence were unable to be reported at the species level owing to some studies not identifying their respective isolated bacteria beyond the genus level. Second, besides non-human sources being excluded from our work just one study came from outside of healthcare settings – though all research settings were eligible for inclusion. This means that the pooled VRE prevalence calculated in our analysis is not an accurate representation of the generalised VRE prevalence in India. Third, several studies utilised in the present investigation isolated and considered *Enterococci* species – namely *E. casseliflavus* and *E. gallinarum* – which exhibit intrinsic resistance to vancomycin as VRE. Therefore, the pooled prevalence calculated in our analysis may be an overestimation of the actual prevalence of acquired VRE in India. Finally, the strength of our investigation was limited by the significant heterogeneity observed between the included studies; such heterogeneity is likely the result of a combination of inconsistencies across the studies, for example varying definitions of VRE alongside methodological differences, which largely reduces study comparability.

## Conclusion

In summary, the current systematic review and meta-analysis provide the most up-to-date evaluation of the rate of VRE infections in India. Our study demonstrated that the pooled prevalence of VRE in India was lower than that of some less developed countries but, in contrast, our finding was higher in comparison to the VRE prevalence estimates of some higher-income countries. Moreover, we observed an increasing trend in VRE prevalence over time in addition to the affirmed presence of VRE in healthy Indian citizens as well as in those suffering from debilitated health. Strict adherence to infection control measures, implementation of a national antimicrobial stewardship programme, and limiting the use of antimicrobials in animal husbandry are all measures required to impede any further rise in AMR – including VRE. Finally, we identified gaps in the present article which should be addressed in future research to expand our knowledge and understanding of VRE prevalence in India.

### Electronic supplementary material

Below is the link to the electronic supplementary material.

Supplementary Material 1

## Data Availability

All data generated or analysed during this study are included in this manuscript.

## Abbreviations

VRE: Vancomycin-resistant *Enterococci*
PRISMA: Preferred Reporting Items for Systematic Reviews and Meta-Analyses
AMR: Antimicrobial resistance
WHO: World Health Organization
UTIs: Urinary tract infections
JBI: Joanna Briggs Institute
AST: Antimicrobial susceptibility testing
MDR: Multidrug resistant

## References

[1] Murray CJL, Ikuta KS, Sharara F, Swetschinski L, Aguilar GR, Gray A (2022). Global burden of bacterial antimicrobial resistance in 2019: a systematic analysis. The Lancet.

[2] Krawczyk B, Wityk P, Gałęcka M, Michalik M (2021). The many faces of Enterococcus spp.—commensal, probiotic and opportunistic pathogen. Microorganisms.

[3] Levy SB, Marshall B (2004). Antibacterial resistance worldwide: causes, challenges and responses. Nat Med.

[4] Weiner LM, Webb AK, Limbago B, Dudeck MA, Patel J, Kallen AJ (2016). Antimicrobial-resistant pathogens associated with healthcare-associated infections: summary of data reported to the National Healthcare Safety Network at the Centers for Disease Control and Prevention, 2011–2014. Infect Control Hosp Epidemiol.

[5] Ott E, Saathoff S, Graf K, Schwab F, Chaberny IF (2013). The prevalence of nosocomial and community acquired infections in a university hospital: an observational study. Dtsch Arzteblatt Int.

[6] Tsai JC, Hsueh PR, Lin HM, Chang HJ, Ho SW, Teng LJ (2005). Identification of clinically relevant *Enterococcus* species by direct sequencing of *groES* and spacer region. J Clin Microbiol.

[7] Dubin K, Pamer EG (2014). Enterococci and their interactions with the intestinal microbiome. Microbiol Spectr.

[8] Miller WR, Munita JM, Arias CA (2014). Mechanisms of antibiotic resistance in *Enterococci*. Expert Rev Anti Infect Ther.

[9] Rubinstein E, Keynan Y (2014). Vancomycin revisited – 60 years later. Front Public Health.

[10] Gold HS (2001). Vancomycin-resistant *Enterococci*: mechanisms and clinical observations. Clin Infect Dis.

[11] Werner G, Coque TM, Hammerum AM, Hope R, Hryniewicz W, Johnson A (2008). Emergence and spread of vancomycin resistance among *Enterococci* in Europe. Euro Surveill.

[12] Leclercq R, Derlot E, Duval J, Courvalin P (1988). Plasmid-mediated resistance to vancomycin and teicoplanin in *Enterococcus faecium*. N Engl J Med.

[13] Uttley AH, George RC, Naidoo J, Woodford N, Johnson AP, Collins CH (1989). High-level vancomycin-resistant *Enterococci* causing hospital infections. Epidemiol Infect.

[14] Balli EP, Venetis CA, Miyakis S (2014). Systematic review and meta-analysis of linezolid versus daptomycin for treatment of vancomycin-resistant enterococcal bacteremia. Antimicrob Agents Chemother.

[15] Kumar SG, Adithan C, Harish BN, Sujatha S, Roy G, Malini A (2013). Antimicrobial resistance in India: a review. J Nat Sci Biol Med.

[16] Taneja N, Sharma M (2019). Antimicrobial resistance in the environment: the indian scenario. Indian J Med Res.

[17] Mathur P, Chaudhary R, Dhawan B, Sharma N, Kumar L (1999). Vancomycin-resistant *Enterococcus* bacteraemia in a lymphoma patient. Indian J Med Microbiol.

[18] Page MJ, McKenzie JE, Bossuyt PM, Boutron I, Hoffmann TC, Mulrow CD (2021). The PRISMA 2020 statement: an updated guideline for reporting systematic reviews. BMJ.

[19] Pearson A, Wiechula R, Court A, Lockwood C (2005). The JBI model of evidence-based healthcare. Int J Evid Based Healthc.

[20] Putnam SD, Sader HS, Moet GJ, Mendes RE, Jones RN (2010). Worldwide summary of telavancin spectrum and potency against Gram-positive pathogens: 2007 to 2008 surveillance results. Diagn Microbiol Infect Dis.

[21] Gordon S, Swenson JM, Hill BC, Pigott NE, Facklam RR, Cooksey RC (1992). Antimicrobial susceptibility patterns of common and unusual species of *Enterococci* causing infections in the United States. Enterococcal Study Group. J Clin Microbiol.

[22] Horner C, Mushtaq S, Allen M, Hope R, Gerver S, Longshaw C (2021). Replacement of Enterococcus faecalis by Enterococcus faecium as the predominant Enterococcus in UK bacteraemias. JAC Antimicrob Resist.

[23] Aun E, Kisand V, Laht M, Telling K, Kalmus P, Väli Ü (2021). Molecular characterization of *Enterococcus* isolates from different sources in Estonia reveals potential transmission of resistance genes among different reservoirs. Front Microbiol.

[24] Hazarika P, Chattopadhyay I, Umpo M, Choudhury Y, Sharma I (2021). Phylogeny, biofilm production, and antimicrobial properties of fecal microbial communities of Adi tribes of Arunachal Pradesh, India. Appl Biochem Biotechnol.

[25] Orababa OQ, Soriwei JD, Akinsuyi SO, Essiet UU, Solesi OM (2021). A systematic review and meta-analysis on the prevalence of vancomycin-resistant *Enterococci* (VRE) among Nigerians. Porto Biomed J.

[26] Melese A, Genet C, Andualem T (2020). Prevalence of vancomycin resistant *enterococci* (VRE) in Ethiopia: a systematic review and meta-analysis. BMC Infect Dis.

[27] Toner L, Papa N, Aliyu SH, Dev H, Lawrentschuk N, Al-Hayek S (2016). Vancomycin resistant *enterococci* in urine cultures: antibiotic susceptibility trends over a decade at a tertiary hospital in the United Kingdom. Investig Clin Urol.

[28] Emaneini M, Hosseinkhani F, Jabalameli F, Nasiri MJ, Dadashi M, Pouriran R (2016). Prevalence of vancomycin-resistant *Enterococcus* in Iran: a systematic review and meta-analysis. Eur J Clin Microbiol Infect Dis.

[29] Shrestha S, Kharel S, Homagain S, Aryal R, Mishra SK (2021). Prevalence of vancomycin-resistant *Enterococci* in Asia-a systematic review and meta-analysis. J Clin Pharm Ther.

[30] Al-Talib H, Zuraina N, Kamarudin B, Yean CY (2015). Genotypic variations of virulent genes in *Enterococcus faecium* and *Enterococcus faecalis* isolated from three hospitals in Malaysia. Adv Clin Exp Med.

[31] Dhandapani S, Rajshekar D, Priyadarshi K, Krishnamoorthi S, Sundaramurthy R, Madigubba H (2023). Comparison of hand hygiene compliance among healthcare workers in intensive care units and wards of COVID-19: a large scale multicentric study in India. Am J Infect Control.

[32] Kritya M, Yadav AK, Shridhar G (2022). A survey of hand hygiene practices among indian medical undergraduates. Med J Armed Forces India.

[33] Banik GR, Durayb B, King C, Rashid H (2022). Antimicrobial resistance following prolonged use of hand hygiene products: a systematic review. Pharmacy.

[34] Larson E (1988). A causal link between handwashing and risk of infection? Examination of the evidence. Infect Control.

[35] Mathur P (2011). Hand hygiene: back to the basics of infection control. Indian J Med Res.

[36] Noskin GA, Stosor V, Cooper I, Peterson LR (1995). Recovery of vancomycin-resistant *Enterococci* on fingertips and environmental surfaces. Infect Control Hosp Epidemiol.

[37] Van Boeckel TP, Gandra S, Ashok A, Caudron Q, Grenfell BT, Levin SA (2014). Global antibiotic consumption 2000 to 2010: an analysis of national pharmaceutical sales data. Lancet Infect Dis.

[38] McGettigan P, Roderick P, Kadam A, Pollock AM (2017). Access, watch, and reserve antibiotics in India: challenges for WHO stewardship. Lancet Glob Health.

[39] Koya SF, Ganesh S, Selvaraj S, Wirtz VJ, Galea S, Rockers PC (2022). Antibiotic consumption in India: geographical variations and temporal changes between 2011 and 2019. JAC Antimicrob Resist.

[40] Kotwani A, Joshi J, Lamkang AS (2021). Over-the-counter sale of antibiotics in India: a qualitative study of providers’ perspectives across two States. Antibiot (Basel).

[41] Kasthuri A (2018). Challenges to healthcare in India - the five A’s. Indian J Community Med.

[42] Blanchard J, Solaipandian M, John EB, Pandith M, Jeo B, Saji S (2021). Self-prescribing of antibiotics by patients seeking care in indian emergency departments. J Am Coll Emerg Physicians Open.

[43] Bose SS, Das M, Barai T, Bhakta S, Das A, Paul S (2021). Usage-evaluation study on vancomycin among hospitalized pediatric patients: experience from two tertiary care centers of Eastern India. Indian J Child Health.

[44] Vijay S, Ramasubramanian V, Bansal N, Ohri VC, Walia K (2023). Hospital-based antimicrobial stewardship, India. Bull World Health Organ.

[45] Pallares C, Hernández-Gómez C, Appel TM, Escandón K, Reyes S, Salcedo S (2022). Impact of antimicrobial stewardship programs on antibiotic consumption and antimicrobial resistance in four colombian healthcare institutions. BMC Infect Dis.

[46] Van Boeckel TP, Brower C, Gilbert M, Grenfell BT, Levin SA, Robinson TP (2015). Global trends in antimicrobial use in food animals. Proc Natl Acad Sci U S A.

[47] Manyi-Loh C, Mamphweli S, Meyer E, Okoh A (2018). Antibiotic use in agriculture and its consequential resistance in environmental sources: potential public health implications. Molecules.

[48] Nilsson O. Vancomycin resistant *enterococci* in farm animals – occurrence and importance. Infect Ecol Epidemiol. 2012;2. 10.3402/iee.v2i0.16959. 10.3402/iee.v2i0.16959 PMC3426332 22957131

[49] Bager F, Madsen M, Christensen J, Aarestrup FM (1997). Avoparcin used as a growth promoter is associated with the occurrence of vancomycin-resistant *Enterococcus faecium* on danish poultry and pig farms. Prev Vet Med.

[50] Bager F, Aarestrup FM, Madsen M, Wegener HC (1999). Glycopeptide resistance in *Enterococcus faecium* from broilers and pigs following discontinued use of avoparcin. Microb Drug Resist.

[51] Klare I, Badstübner D, Konstabel C, Böhme G, Claus H, Witte W (1999). Decreased incidence of VanA-type vancomycin-resistant *Enterococci* isolated from poultry meat and from fecal samples of humans in the community after discontinuation of avoparcin usage in animal husbandry. Microb Drug Resist.

[52] Borah D, Singh V, Gogoi B, Hazarika M, Rahman A (2016). Prevalence of multidrug resistant (MDR) novel *Enterococcus faecium* strain VDR03 in broiler chicken meat samples collected from Dibrugarh town, Assam (India). Res J Microbiol.

[53] Soni MM, Nayak JB, Anjaria PA, Bhavsar PP, Chaudhary JH, Brahmbhatt MN (2022). Isolation and molecular detection of biofilm producing and multiple drug resistant *Enterococcus faecalis* from the buffalo meat. Buffalo Bull.

[54] Preethi C, Thumu SCR, Halami PM (2017). Occurrence and distribution of multiple antibiotic-resistant *Enterococcus* and *Lactobacillus* spp. from indian poultry: in vivo transferability of their erythromycin, tetracycline and vancomycin resistance. Ann Microbiol.

[55] Chaudhry D, Tomar P (2017). Antimicrobial resistance: the next BIG pandemic. Int J Community Med Public Health.

[56] Bhargava K, Nath G, Bhargava A, Kumari R, Aseri GK, Jain N (2022). Bacterial profile and antibiotic susceptibility pattern of uropathogens causing urinary tract infection in the eastern part of Northern India. Front Microbiol.

[57] Bhatt P, Patel A, Sahni AK, Praharaj AK, Grover N, Chaudhari CN (2015). Emergence of multidrug resistant *enterococci* at a tertiary care centre. Med J Armed Forces India.

[58] Das AK, Dudeja M, Kohli S, Ray P (2022). Genotypic characterization of vancomycin-resistant *Enterococcus* causing urinary tract infection in Northern India. Indian J Med Res.

[59] Das S, Konar J, Talukdar M (2021). Prevalence of vancomycin-resistant *Enterococcus* causing urinary tract infection in a tertiary care hospital of Eastern India. Biomed Biotechnol Res J.

[60] Deshpande VR, Karmarkar MG, Mehta PR (2013). Prevalence of multidrug-resistant *Enterococci* in a tertiary care hospital in Mumbai, India. J Infect Dev Ctries.

[61] Gangurde N, Mane M, Phatale S (2014). Prevalence of multidrug resistant *Enterococci* in a tertiary care hospital in India: a growing threat. Open J Med Microbiol.

[62] Goel V, Kumar D, Kumar R, Mathur P, Singh S (2016). Community acquired enterococcal urinary tract infections and antibiotic resistance profile in North India. J Lab Physicians.

[63] Jain AK, Patidar H, Nayak V, Agrawal R (2022). Prevalence, risk factors and microbial profile of surgical site infection after cesarean section in a tertiary care center in western India. J Pure Appl Microbiol.

[64] Kapoor L, Randhawa VS, Deb M (2005). Antimicrobial resistance of enterococcal blood isolates at a pediatric care hospital in India. Jpn J Infect Dis.

[65] Meena S, Mohapatra S, Sood S, Dhawan B, Das BK, Kapil A (2017). Revisiting nitrofurantoin for vancomycin resistant *enterococci*. J Clin Diagn Res.

[66] Phukan C, Lahkar M, Ranotkar S, Saikia KK (2016). Emergence of *vanA* gene among vancomycin-resistant *Enterococci* in a tertiary care hospital of North - East India. Indian J Med Res.

[67] Praharaj I, Sujatha S, Parija SC (2013). Phenotypic & genotypic characterization of vancomycin resistant *Enterococcus* isolates from clinical specimens. Indian J Med Res.

[68] Purohit G, Gaind R, Dawar R, Verma PK, Aggarwal KC, Sardana R (2017). Characterization of vancomycin resistant *enterococci* in hospitalized patients and role of gut colonization. J Clin Diagn Res.

[69] Sami H, Singh A, Ahmed S, Shahid M (2020). Emergence of linezolid resistance in *Enterococci*: prevalent genotypes and resistance pattern in vancomycin-resistant *Enterococci* in a north-indian tertiary care hospital. N Z J Med Lab Sci.

[70] Shinde RS, Koppikar GV, Oommen S (2012). Characterization and antimicrobial susceptibility pattern of clinical isolates of *Enterococci* at a tertiary care hospital in Mumbai, India. Ann Trop Med Public Health.

[71] Sreeja S, Babu PRS, Prathab AG (2012). The prevalence and the characterization of the *Enterococcus* species from various clinical samples in a tertiary care hospital. J Clin Diagn Res.

[72] Taneja N, Rani P, Emmanuel R, Sharma M (2004). Significance of vancomycin resistant *enterococci* from urinary specimens at a tertiary care centre in northern India. Indian J Med Res.

[73] Yadav RK, Agarwal L (2022). *Enterococcal* infections in a tertiary care hospital, North India. Ann Afr Med.

