# Supplementary material for: Prevalence of vancomycin-resistant Enterococci in India between 2000 and 2022: a systematic review and meta-analysis
Source: Antimicrob Resist Infect Control. 2023 Aug 21;12:79. doi: 10.1186/s13756-023-01287-z (PMC10441759; doi:10.1186/s13756-023-01287-z)
Supplement: Supplementary file 1 — Supplementary Material 1 [file 13756_2023_1287_MOESM1_ESM.pdf]

SUPPLEMENTARY INFORMATION

Prevalence of vancomycin-resistant *Enterococci* in India between 2000 and 2022: A systematic review and meta-analysis

Emily Smout[1], Navaneethan Palanisamy[1], Sabeel P Valappil[1]

[1]Chester Medical School, University of Chester, Bache Hall, Countess View, Chester CH2 1BR, UK

Corresponding authors:

Navaneethan Palanisamy (E-mail: n.palanisamy@chester.ac.uk)

Sabeel P Valappil (E-mail: s.valappil@chester.ac.uk)

Supplementary Table 1 Exact database search inputs

PubMed	(((((((Enterococc*) OR (Enterococcus faecalis)) OR (Enterococcus faecium)) OR (E. faecalis)) OR (E. faecium)) AND (((((Vancomycin resistan*) OR (Antibiotic resistan*)) OR (Antimicrobial resistan*)) OR (Drug resistan*)) OR (VRE))) AND ((((((Epidemiology) OR (prevalence)) OR (rate)) OR (frequency)) OR (Cross-sectional stud*)) OR (Epidemiologic stud*))) AND India
Scopus	TITLE-ABS-KEY ((((((((enterococc*) OR (enterococcus AND faecalis)) OR (enterococcus AND faecium)) OR (e. AND faecalis)) OR (e. AND faecium)) AND (((((vancomycin AND resistan*) OR (antibiotic AND resistan*)) OR (antimicrobial AND resistan*)) OR (drug AND resistan*)) OR (vre))) AND ((((((epidemiology) OR (prevalence)) OR (rate)) OR (frequency)) OR (cross-sectional AND stud*)) OR (epidemiologic AND stud*))) AND India)
Google Scholar	Prevalence | frequency | rate + Enterococci | Enterococcus | Enterococcus faecalis | Enterococcus faecium | E. faecalis | E. faecium + Vancomycin resistan* | Antibiotic resistan* | Antimicrobial resistan* | Drug resistan* | VRE + India -Iran -Indonesia

Supplementary Table 2 Quality assessment of studies using Joanna Briggs Institute (JBI) critical appraisal checklist for studies reporting prevalence data

Study name	Checklists*									Total score
	1	2	3	4	5	6	7	8	9	
Bhargava et al. 2022 [1]	Yes	Yes	Yes	Yes	Unclear	Yes	Yes	Yes	Yes	8
Bhatt et al. 2015 [2]	Yes	Yes	Yes	Yes	Yes	Yes	Yes	Unclear	Yes	8
Das et al. 2022 [3]	Yes	Yes	Yes	Unclear	Unclear	Yes	Yes	Yes	Yes	7
Das et al. 2021 [4]	Yes	Yes	Yes	Yes	Yes	Yes	Yes	Yes	Yes	9
Deshpande et al. 2013 [5]	Yes	Yes	Yes	No	Unclear	Yes	Yes	Yes	Yes	7
Gangurde et al. 2014 [6]	Yes	Yes	Yes	Unclear	Unclear	Yes	Yes	Unclear	Yes	6
Goel et al. 2016 [7]	Yes	Yes	Yes	Yes	Yes	Yes	Yes	Unclear	Yes	8
Hazarika et al. 2021 [8]	Yes	Unclear	No	Yes	No	Yes	Yes	Yes	Yes	6
Jain et al. 2022 [9]	Yes	Yes	Yes	Yes	Yes	Yes	Yes	Yes	Yes	9
Kapoor et al. 2005 [10]	Yes	Yes	Unclear	Yes	Yes	Yes	Yes	Unclear	Yes	7
Meena et al. 2017 [11]	Yes	Yes	Yes	Yes	Yes	Yes	Yes	Yes	Yes	9

Phukan et al. 2016 [12]	Yes	Yes	Yes	No	Unclear	Yes	Yes	Unclear	Yes	6
Praharaj et al. 2013 [13]	Yes	Yes	Yes	No	Unclear	Yes	Yes	Yes	Yes	7
Purohit et al. 2017 [14]	Yes	Yes	Yes	Unclear	Unclear	Yes	Yes	Yes	Yes	7
Sami et al. 2020 [15]	Yes	Yes	Yes	Yes	Yes	Yes	Yes	Unclear	Yes	8
Shinde et al. 2012 [16]	Yes	Yes	Yes	No	No	Yes	Yes	Unclear	Yes	6
Sreeja et al. 2012 [17]	Yes	Yes	Yes	No	Unclear	Yes	Yes	Unclear	Yes	6
Taneja et al. 2004 [18]	Yes	Yes	Yes	No	Unclear	Yes	Yes	No	Yes	6
Yadav & Agarwal 2022 [19]	Yes	Yes	Yes	Unclear	No	Yes	Yes	Unclear	Yes	6

Note: *Checklists were as follows: 1. Was the sample frame appropriate to address the target population? 2. Were study participants sampled in an appropriate way? 3. Was the sample size adequate? 4. Were the study subjects and the setting described in detail? 5. Was the data analysis conducted with sufficient coverage of the identified sample? 6. Were valid methods used for the identification of the condition? 7. Was the condition measured in a standard, reliable way for all participants? 8. Was there appropriate statistical analysis? 9. Was the response rate adequate, and if not, was the low response rate managed appropriately?

Supplementary Table 3 *Enterococci* species isolated in the included studies

Study	Total *Enterococci* isolates	*Enterococci* species isolated	Number of other *Enterococci* isolates* (n=)	Number of other VRE species* (n=)
Bhargava et al. 2022 [1]	18	-	-	-
Bhatt et al. 2015 [2]	200	*E. faecalis*, *E. faecium*	0	0
Das et al. 2022 [3]	118	*E. avium*, *E. durans*, *E. faecalis*, *E. faecium*, *E. gallinarum*	*E. avium* (2), *E. durans* (1), *E. gallinarum* (1)	0
Das et al. 2021 [4]	457	*E. faecalis*, *E. faecium*	0	0

Deshpande et al. 2013 [5]	291	*E. faecalis*,<br>*E. faecium*	0	0
Gangurde et al. 2014 [6]	180	*E. durans*,<br>*E. faecalis*,<br>*E. faecium*,<br>*E. raffinosus*	*E. durans* (8),<br>*E. raffinosus* (6)	*E. durans* (1),<br>*E. raffinosus* (1)
Goel et al. 2016 [7]	115	*E. avium*,<br>*E. casseliflavus*,<br>*E. dispar*,<br>*E. faecalis*,<br>*E. faecium*,<br>*E. gallinarum*,<br>*E. pseudoavium*	*E. avium* (2),<br>*E. casseliflavus* (2),<br>*E. dispar* (3),<br>*E. gallinarum* (2),<br>*E. pseudoavium* (3)	*E. gallinarum* (2)
Hazarika et al. 2021 [8]	6	-	-	-
Jain et al. 2022 [9]	9	-	-	-

Kapoor et al. 2005 [10]	50	*E. dispar*, *E. durans*, *E. faecalis*, *E. faecium*	*E. dispar* (3), *E. durans* (4)	0
Meena et al. 2017 [11]	70	*E. faecalis*, *E. faecium*	0	0
Phukan et al. 2016 [12]	67	*E. faecalis*, *E. faecium*	0	0
Praharaj et al. 2013 [13]	367	*E. faecalis*, *E. gallinarum*, *E. mundtii*	-	*E. gallinarum* (2), *E. mundtii* (1)
Purohit et al. 2017 [14]	250	*E. faecalis*, *E. faecium*, *E. gallinarum*	*E. gallinarum* (6)	0
Sami et al. 2020 [15]	1014	*E. faecalis*, *E. faecium*	0	0

Shinde et al. 2012 [16]	54	*E. durans*, *E. faecalis*, *E. faecium*	*E. durans* (2)	0
Sreeja et al. 2012 [17]	128	*E. faecalis*, *E. faecium*	0	0
Taneja et al. 2004 [18]	144	*E. avium*, *E. casseliflavus*, *E. faecalis*, *E. faecium*, *E. mundtii*, *E. pseudoavium*	*E. avium* (5), *E. casseliflavus* (34), *E. mundtii* (5), *E. pseudoavium* (3)	*E. casseliflavus* (1), *E. pseudoavium* (1)
Yadav & Agarwal 2022 [19]	145	*E. durans*, *E. faecalis*, *E. faecium*, *E. gallinarum*	*E. durans* (2), *E. gallinarum* (1)	*E. durans* (1)

Note: '-' means not given, '*' means *Enterococci* species other than *E. faecalis* and *E. faecium*. VRE = vancomycin-resistant *Enterococci*

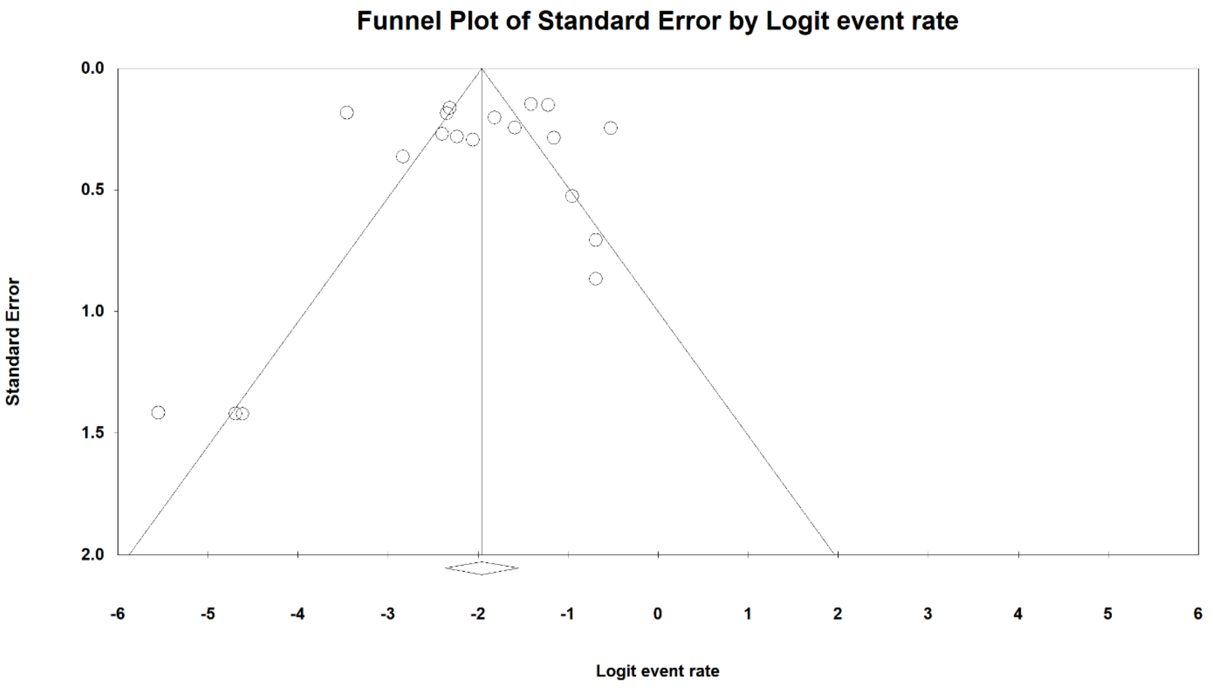

Supplementary Figure 1 Funnel plot to observe publication bias before applying trim-and-fill method.

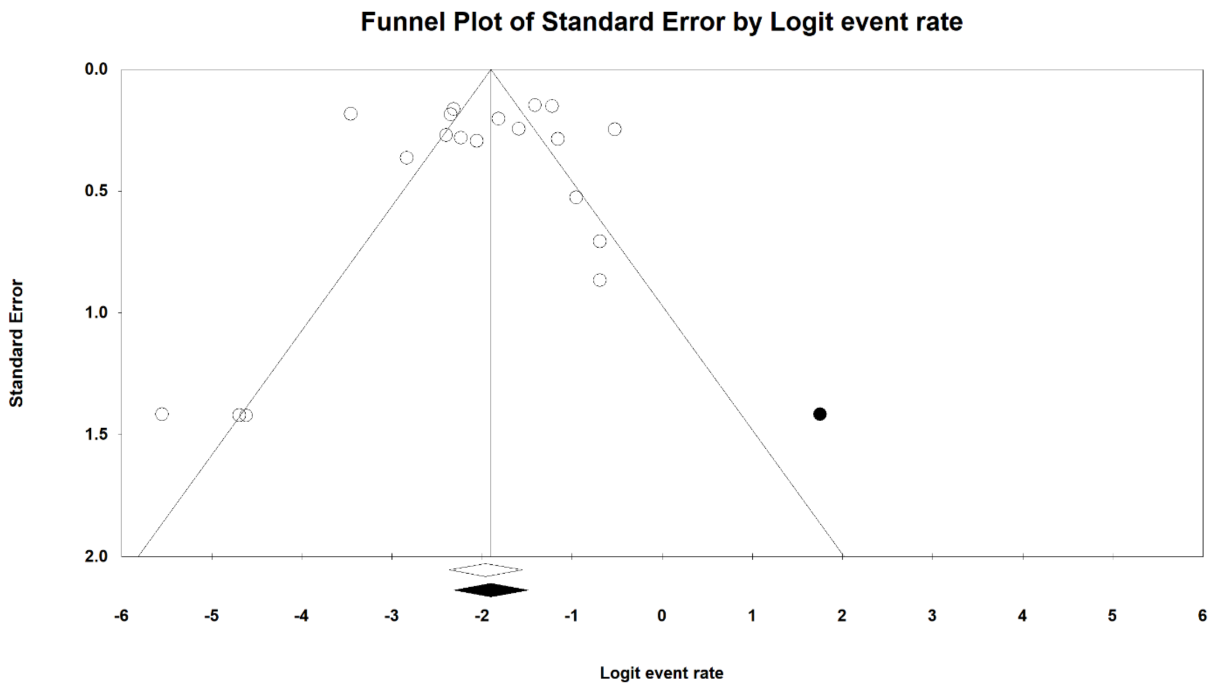

Supplementary Figure 2 Funnel plot to observe publication bias after applying trim-and-fill method.

References

1. Bhargava K, Nath G, Bhargava A, Kumari R, Aseri GK, Jain N. Bacterial profile and antibiotic susceptibility pattern of uropathogens causing urinary tract infection in the eastern part of Northern India. Front Microbiol. 2022;13:965053. doi: 10.3389/fmicb.2022.965053

2. Bhatt P, Patel A, Sahni AK, Praharaj AK, Grover N, Chaudhari CN, et al. Emergence of multidrug resistant *Enterococci* at a tertiary care centre. Med J Armed Forces India. 2015;71:139–44. doi: 10.1016/j.mjafi.2014.08.007

3. Das AK, Dudeja M, Kohli S, Ray P. Genotypic characterization of vancomycin-resistant *Enterococcus* causing urinary tract infection in Northern India. Indian J Med Res. 2022;155:423–31. doi: 10.4103/ijmr.IJMR_2554_19

4. Das S, Konar J, Talukdar M. Prevalence of vancomycin-resistant *Enterococcus* causing urinary tract infection in a tertiary care hospital of Eastern India. Biomed Biotechnol Res J. 2021;5:463–5. doi: 10.4103/bbrj.bbrj_212_21

5. Deshpande VR, Karmarkar MG, Mehta PR. Prevalence of multidrug-resistant *Enterococci* in a tertiary care hospital in Mumbai, India. J Infect Dev Ctries. 2013;7:155–8. doi: 10.3855/jidc.3018

6. Gangurde N, Mane M, Phatale S. Prevalence of multidrug resistant *Enterococci* in a tertiary care hospital in India: a growing threat. Open J Med Microbiol. 2014;4:11–5. doi: 10.4236/ojmm.2014.41002

7. Goel V, Kumar D, Kumar R, Mathur P, Singh S. Community acquired Enterococcal urinary tract infections and antibiotic resistance profile in North India. J Lab Physicians. 2016;8:50–4. doi: 10.4103/0974-2727.176237

8. Hazarika P, Chattopadhyay I, Umpo M, Choudhury Y, Sharma I. Phylogeny, biofilm production, and antimicrobial properties of fecal microbial communities of Adi tribes of Arunachal Pradesh, India. Appl Biochem Biotechnol. 2021;193:1675–87. doi: 10.1007/s12010-021-03535-7

9. Jain AK, Patidar H, Nayak V, Agrawal R. Prevalence, risk factors and microbial profile of surgical site infection after cesarean section in a tertiary care center in Western India. J Pure Appl Microbiol. 2022;16:700–7. doi: 10.22207/JPAM.16.1.73

10. Kapoor L, Randhawa VS, Deb M. Antimicrobial resistance of Enterococcal blood isolates at a pediatric care hospital in India. Jpn J Infect Dis. 2005;58:101–3.

11. Meena S, Mohapatra S, Sood S, Dhawan B, Das BK, Kapil A. Revisiting nitrofurantoin for vancomycin resistant *Enterococci*. J Clin Diagn Res. 2017;11:DC19–22. doi: 10.7860/JCDR/2017/25140.10140

12. Phukan C, Lahkar M, Ranotkar S, Saikia KK. Emergence of *vanA* gene among vancomycin-resistant *Enterococci* in a tertiary care hospital of North - East India. Indian J Med Res. 2016;143:357–61. doi: 10.4103/0971-5916.182627

13. Praharaj I, Sujatha S, Parija SC. Phenotypic & genotypic characterization of vancomycin resistant *Enterococcus* isolates from clinical specimens. Indian J Med Res. 2013;138:549–56.

14. Purohit G, Gaind R, Dawar R, Verma PK, Aggarwal KC, Sardana R, et al. Characterization of vancomycin resistant *Enterococci* in hospitalized patients and role of gut colonization. J Clin Diagn Res. 2017;11:DC01-05. doi: 10.7860/JCDR/2017/25988.10548

15. Sami H, Singh A, Ahmed S, Shahid M. Emergence of linezolid resistance in *Enterococci*: prevalent genotypes and resistance pattern in vancomycin-resistant *Enterococci* in a North-Indian tertiary care hospital. N Z J Med Lab Sci. 2020;74:27–30. doi: 10.3316/informit.323077107272382

16. Shinde RS, Koppikar GV, Oommen S. Characterization and antimicrobial susceptibility pattern of clinical isolates of *Enterococci* at a tertiary care hospital in Mumbai, India. Ann Trop Med Public Health. 2012;5:85–8. doi: 10.4103/1755-6783.95956

17. Sreeja S, Babu PRS, Prathab AG. The prevalence and the characterization of the *Enterococcus* species from various clinical samples in a tertiary care hospital. J Clin Diagn Res. 2012;6:1486–8. doi: 10.7860/JCDR/2012/4560.2539

18. Taneja N, Rani P, Emmanuel R, Sharma M. Significance of vancomycin resistant *Enterococci* from urinary specimens at a tertiary care centre in Northern India. Indian J Med Res. 2004;119:72–4.

19. Yadav RK, Agarwal L. Enterococcal infections in a tertiary care hospital, North India. Ann Afr Med. 2022;21:193–7. doi: 10.4103/aam.aam_110_20
